# A second triclinic polymorph of bis­(μ-*N*-nitroso-*N*-phenyl­hydroxy­laminato)-κ^3^ *O*,*O*′:*O*′;κ^3^ *O*′:*O*,*O*′-bis­[(*N*-nitroso-*N*-phenyl­hydroxy­laminato-κ^2^ *O*,*O*′)lead(II)]

**DOI:** 10.1107/S1600536812021885

**Published:** 2012-05-26

**Authors:** Ezzatollah Najafi, Mostafa M. Amini, Seik Weng Ng

**Affiliations:** a Department of Chemistry, General Campus, Shahid Beheshti University, Tehran 1983963113, Iran; b Department of Chemistry, University of Malaya, 50603 Kuala Lumpur, Malaysia; c Chemistry Department, Faculty of Science, King Abdulaziz University, PO Box 80203 Jeddah, Saudi Arabia

## Abstract

The cupferronate ions in the centrosymmetric dinuclear title compound, [Pb_2_(C_6_H_5_N_2_O_2_)_4_], *O*,*O*′-chelate to the two Pb^II^ atoms; two of the four nitroso O atoms are also involved in bridging. The geometries of the five-coordinate Pb^II^ atoms in the two independent mol­ecules are Ψ-octa­hedral; if more remote Pb⋯O inter­actions are also considered, the coordination number is increased to six for one mol­ecule and to seven for the other. Their coordination polyhedra are ill defined in the chain motif, which runs along [100].

## Related literature

For the first triclinic polymorph, see: Najafi *et al.* (2011 ▶).
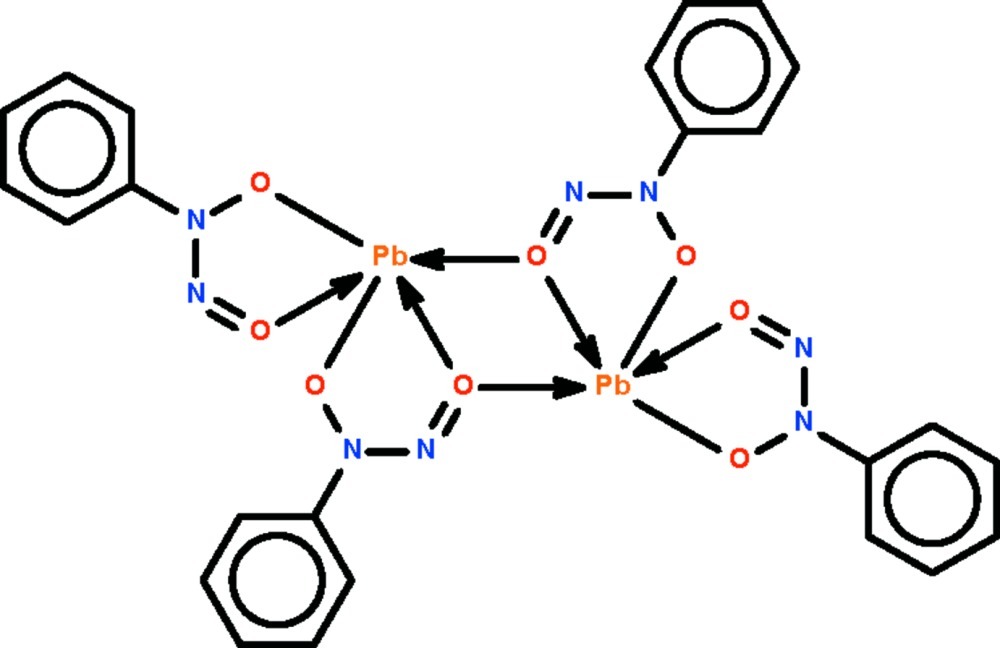


## Experimental

### 

#### Crystal data

[Pb_2_(C_6_H_5_N_2_O_2_)_4_]
*M* *_r_* = 962.86
Triclinic, 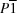

*a* = 8.7579 (2) Å
*b* = 10.6985 (3) Å
*c* = 15.3603 (6) Å
α = 72.079 (3)°
β = 77.582 (3)°
γ = 86.538 (2)°
*V* = 1337.31 (7) Å^3^
*Z* = 2
Mo *K*α radiation
μ = 12.64 mm^−1^
*T* = 100 K
0.25 × 0.20 × 0.15 mm

#### Data collection

Agilent SuperNova Dual diffractometer with an Atlas detector
Absorption correction: multi-scan (*CrysAlis PRO*; Agilent, 2012) ▶ *T*_min_ = 0.144, *T*_max_ = 0.253
20132 measured reflections
6173 independent reflections
5479 reflections with *I* > 2σ(*I*)
*R*_int_ = 0.040

#### Refinement

*R*[*F* ^2^ > 2σ(*F* ^2^)] = 0.024
*wR*(*F* ^2^) = 0.057
*S* = 1.04
6173 reflections
379 parameters
H-atom parameters constrained
Δρ_max_ = 0.78 e Å^−3^
Δρ_min_ = −1.67 e Å^−3^

### 

Data collection: *CrysAlis PRO* (Agilent, 2012 ▶); cell refinement: *CrysAlis PRO*; data reduction: *CrysAlis PRO*; program(s) used to solve structure: *SHELXS97* (Sheldrick, 2008 ▶); program(s) used to refine structure: *SHELXL97* (Sheldrick, 2008 ▶); molecular graphics: *X-SEED* (Barbour, 2001 ▶); software used to prepare material for publication: *publCIF* (Westrip, 2010 ▶).

## Supplementary Material

Crystal structure: contains datablock(s) global, I. DOI: 10.1107/S1600536812021885/lh5471sup1.cif

Structure factors: contains datablock(s) I. DOI: 10.1107/S1600536812021885/lh5471Isup2.hkl

Additional supplementary materials:  crystallographic information; 3D view; checkCIF report

## Figures and Tables

**Table 1:** Selected bond lengths (Å)

Pb1—O1	2.464 (3)
Pb1—O1^i^	2.599 (3)
Pb1—O2	2.475 (3)
Pb1—O3	2.341 (3)
Pb1—O4	2.410 (3)
Pb2—O5	2.385 (3)
Pb2—O6	2.446 (3)
Pb2—O7	2.393 (3)
Pb2—O7^ii^	2.943 (3)
Pb2—O8	2.340 (3)

Symmetry codes: (i) 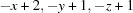; (ii) 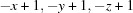.

**Fig. 1.**
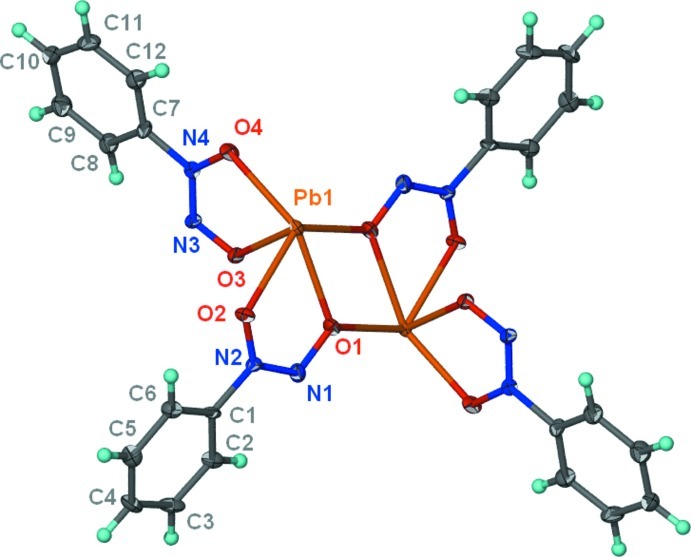
Thermal ellipsoid plot (Barbour, 2001) of one independent [Pb(C6H5N2O2)2]2 molecule at the 70% probability level; hydrogen atoms are drawn as spheres of arbitrary radius. Symmetry-related atoms are not labeled.

**Fig. 2.**
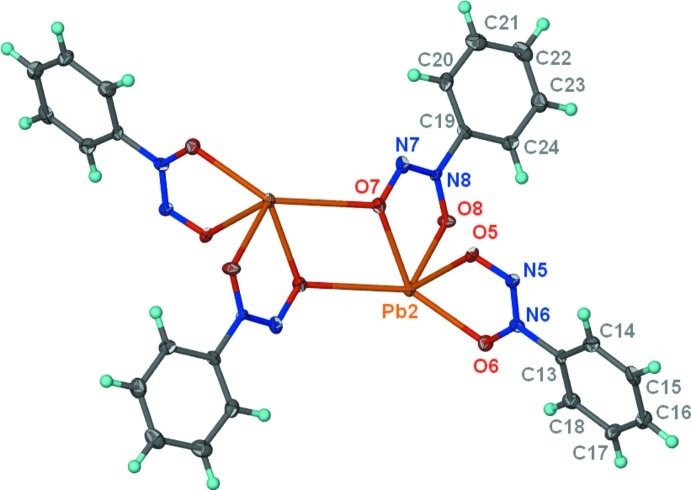
Thermal ellipsoid plot (Barbour, 2001) of second independent [Pb(C6H5N2O2)2]2 molecule at the 70% probability level; hydrogen atoms are drawn as spheres of arbitrary radius. Symmetry-related atoms are not labeled.

**Fig. 3.**
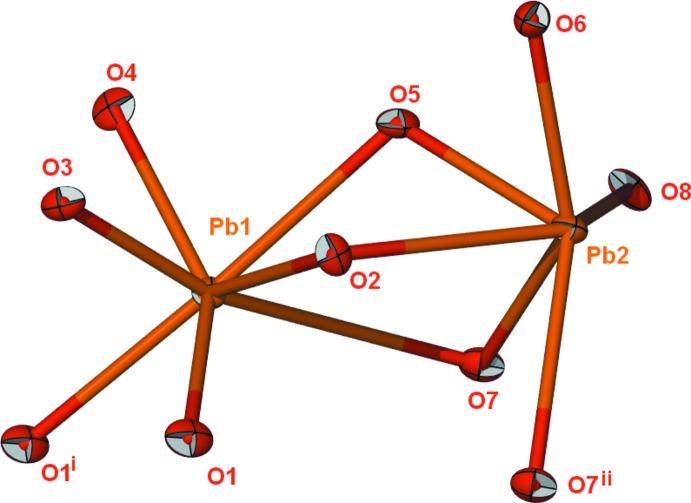
Geometry of PbII in the two independent molecules.

## supplementary crystallographic information

### Comment

In dinuclear [Pb(C_6_H_5_N_2_O_2_)_2_]_2_, the two cupferronate ions chelate to the each of the two Pb_II_ atoms; two of the four nitroso O atoms are also involved in bridging. The geometry of both independent five-coordinate Pb^II^atoms is distorted *Ψ*-octahedron; if another two longer intermolecular Pb··· interactions [at 2.955 (1) and 3.099 (1) Å] are considered, the geometry is a distorted *Ψ*-square antiprism (Najafi *et al.*, 2011). In the second polymorph, which also crystallizes in the triclinic unit cell setting (Scheme I), the two independent dinuclear [Pb_2_(C_6_H_5_N_2_O_2_)_4_] molecules lie on centers-of-inversion. The anion *O*,*O*'-chelates to the Pb^II^ atoms; one of the two nitroso O atoms is also involved in bridging. The bridge in one dimeric molecule is of a normal length [Pb–O 2.599 (3) Å] compared with that in the other [Pb–O 2.943 (3) Å] (Table 1).

The geometry of the five-coordinate Pb^II^ atom in the two independent molecules is *Ψ*-octahedral. For the dimer with the Pb1 atom, if two other Pb···O interactions are considered [Pb1–O5 2.761 (3), Pb1–O7 3.168 (3) Å], the coordination number is increased to seven.

For the dimer with the Pb2 atom, an additional interaction [Pb2···O2 2.843 (3) Å] raises the coordination number to six. This interaction is, however, shorter than the bridging interaction [Pb2–O7^ii^ 2.943 (3) Å].

### Experimental

Lead(II) nitrate (0.33 g, 1 mmol) dissolved in ethanol (20 ml) was added to a solution of the cupferron ligand (0.31 g, 2 mmol) and pyrazine (0.08 g, 1 mmol) dissolved in ethanol (20 ml). The mixture was stirred and then set aside for the growth of brown colored crystals. The *N*-heterocycle was not incorporated in the final product.

### Refinement

Carbon-bound H-atoms were placed in calculated positions [C–H 0.95 Å, *U*_iso_(H) 1.2*U*_eq_(C)] and were included in the refinement in the riding model approximation.

The final difference Fourier map had a peak at 1.30 Å from Pb2 and a hole at 0.70 Å from Pb1.

### Crystal data

**Table:**

[Pb_2_(C_6_H_5_N_2_O_2_)_4_]	*Z* = 2
*M**_r_* = 962.86	*F*(000) = 896
Triclinic, *P*1	*D*_x_ = 2.391 Mg m^−^^3^
Hall symbol: -P 1	Mo *K*α radiation, λ = 0.71073 Å
*a* = 8.7579 (2) Å	Cell parameters from 11683 reflections
*b* = 10.6985 (3) Å	θ = 2.4–27.5°
*c* = 15.3603 (6) Å	µ = 12.64 mm^−^^1^
α = 72.079 (3)°	*T* = 100 K
β = 77.582 (3)°	Prism, light brown
γ = 86.538 (2)°	0.25 × 0.20 × 0.15 mm
*V* = 1337.31 (7) Å^3^	

### Data collection

**Table:**

Agilent SuperNova Dual diffractometer with an Atlas detector	6173 independent reflections
Radiation source: SuperNova (Mo) X-ray Source	5479 reflections with *I* > 2σ(*I*)
Mirror monochromator	*R*_int_ = 0.040
Detector resolution: 10.4041 pixels mm^-1^	θ_max_ = 27.6°, θ_min_ = 2.4°
ω scan	*h* = −11→11
Absorption correction: multi-scan (*CrysAlis PRO*; Agilent, 2012)	*k* = −13→13
*T*_min_ = 0.144, *T*_max_ = 0.253	*l* = −19→19
20132 measured reflections	

### Refinement

**Table:**

Refinement on *F*^2^	Primary atom site location: structure-invariant direct methods
Least-squares matrix: full	Secondary atom site location: difference Fourier map
*R*[*F*^2^ > 2σ(*F*^2^)] = 0.024	Hydrogen site location: inferred from neighbouring sites
*wR*(*F*^2^) = 0.057	H-atom parameters constrained
*S* = 1.04	*w* = 1/[σ^2^(*F*_o_^2^) + (0.0248*P*)^2^] where *P* = (*F*_o_^2^ + 2*F*_c_^2^)/3
6173 reflections	(Δ/σ)_max_ = 0.001
379 parameters	Δρ_max_ = 0.78 e Å^−^^3^
0 restraints	Δρ_min_ = −1.67 e Å^−^^3^

### Fractional atomic coordinates and isotropic or equivalent isotropic displacement parameters (Å^2^)

**Table:**

	*x*	*y*	*z*	*U*_iso_*/*U*_eq_	
Pb1	0.888148 (16)	0.622909 (14)	0.393469 (10)	0.00983 (5)	
Pb2	0.471808 (16)	0.531765 (14)	0.351907 (10)	0.01053 (5)	
O1	0.9179 (3)	0.3990 (3)	0.4962 (2)	0.0156 (6)	
O2	0.7803 (3)	0.4374 (3)	0.3609 (2)	0.0125 (6)	
O3	1.1137 (3)	0.5409 (3)	0.3170 (2)	0.0129 (6)	
O4	1.0296 (3)	0.7788 (3)	0.2542 (2)	0.0141 (6)	
O5	0.6877 (3)	0.6768 (3)	0.2704 (2)	0.0133 (6)	
O6	0.5766 (3)	0.5392 (3)	0.1894 (2)	0.0131 (6)	
O7	0.5208 (3)	0.6402 (3)	0.4582 (2)	0.0139 (6)	
O8	0.3623 (3)	0.7412 (3)	0.3313 (2)	0.0143 (6)	
N1	0.9072 (4)	0.2972 (3)	0.4689 (2)	0.0140 (7)	
N2	0.8377 (4)	0.3219 (3)	0.3994 (2)	0.0101 (7)	
N3	1.1933 (4)	0.6107 (3)	0.2353 (2)	0.0115 (7)	
N4	1.1462 (4)	0.7288 (3)	0.2075 (2)	0.0098 (7)	
N5	0.7635 (4)	0.6830 (3)	0.1859 (2)	0.0127 (7)	
N6	0.7028 (4)	0.6103 (3)	0.1488 (2)	0.0110 (7)	
N7	0.4850 (4)	0.7643 (3)	0.4436 (2)	0.0131 (7)	
N8	0.4051 (4)	0.8099 (3)	0.3794 (2)	0.0086 (7)	
C1	0.8242 (4)	0.2155 (4)	0.3612 (3)	0.0112 (8)	
C2	0.9125 (5)	0.1027 (4)	0.3863 (3)	0.0148 (9)	
H2	0.9813	0.0941	0.4279	0.018*	
C3	0.8967 (5)	0.0026 (4)	0.3484 (3)	0.0176 (10)	
H3	0.9558	−0.0755	0.3645	0.021*	
C4	0.7972 (5)	0.0148 (4)	0.2881 (3)	0.0171 (9)	
H4	0.7874	−0.0545	0.2631	0.021*	
C5	0.7117 (5)	0.1286 (4)	0.2640 (3)	0.0171 (9)	
H5	0.6422	0.1368	0.2228	0.020*	
C6	0.7265 (5)	0.2315 (4)	0.2996 (3)	0.0146 (9)	
H6	0.6703	0.3108	0.2816	0.018*	
C7	1.2235 (5)	0.8123 (4)	0.1153 (3)	0.0131 (9)	
C8	1.3608 (5)	0.7689 (4)	0.0693 (3)	0.0166 (9)	
H8	1.4080	0.6888	0.0979	0.020*	
C9	1.4266 (5)	0.8479 (4)	−0.0206 (3)	0.0195 (10)	
H9	1.5200	0.8210	−0.0544	0.023*	
C10	1.3564 (5)	0.9660 (4)	−0.0612 (3)	0.0183 (9)	
H10	1.4008	1.0184	−0.1228	0.022*	
C11	1.2230 (5)	1.0060 (4)	−0.0118 (3)	0.0192 (10)	
H11	1.1767	1.0872	−0.0392	0.023*	
C12	1.1545 (5)	0.9295 (4)	0.0777 (3)	0.0165 (9)	
H12	1.0625	0.9575	0.1120	0.020*	
C13	0.7777 (4)	0.6118 (4)	0.0551 (3)	0.0098 (8)	
C14	0.9238 (5)	0.6690 (4)	0.0137 (3)	0.0149 (9)	
H14	0.9764	0.7090	0.0464	0.018*	
C15	0.9922 (5)	0.6669 (4)	−0.0762 (3)	0.0165 (9)	
H15	1.0919	0.7065	−0.1055	0.020*	
C16	0.9158 (5)	0.6075 (4)	−0.1235 (3)	0.0173 (9)	
H16	0.9637	0.6053	−0.1847	0.021*	
C17	0.7699 (5)	0.5515 (4)	−0.0815 (3)	0.0159 (9)	
H17	0.7169	0.5121	−0.1144	0.019*	
C18	0.7002 (4)	0.5525 (4)	0.0084 (3)	0.0153 (9)	
H18	0.6005	0.5129	0.0376	0.018*	
C19	0.3613 (4)	0.9468 (4)	0.3584 (3)	0.0112 (8)	
C20	0.3630 (4)	1.0144 (4)	0.4218 (3)	0.0143 (9)	
H20	0.3882	0.9716	0.4809	0.017*	
C21	0.3265 (5)	1.1477 (4)	0.3959 (3)	0.0192 (10)	
H21	0.3283	1.1967	0.4378	0.023*	
C22	0.2877 (5)	1.2099 (4)	0.3100 (3)	0.0191 (10)	
H22	0.2637	1.3009	0.2930	0.023*	
C23	0.2842 (5)	1.1385 (4)	0.2494 (3)	0.0176 (9)	
H23	0.2563	1.1805	0.1909	0.021*	
C24	0.3211 (4)	1.0058 (4)	0.2730 (3)	0.0135 (9)	
H24	0.3187	0.9567	0.2312	0.016*	

### Atomic displacement parameters (Å^2^)

**Table:**

	*U*^11^	*U*^22^	*U*^33^	*U*^12^	*U*^13^	*U*^23^
Pb1	0.01166 (8)	0.00969 (8)	0.00971 (9)	0.00014 (6)	−0.00231 (6)	−0.00513 (6)
Pb2	0.01357 (8)	0.00947 (8)	0.00833 (9)	−0.00020 (6)	−0.00354 (6)	−0.00146 (6)
O1	0.0233 (16)	0.0126 (15)	0.0133 (16)	−0.0039 (12)	−0.0048 (13)	−0.0059 (13)
O2	0.0136 (14)	0.0086 (14)	0.0169 (16)	0.0002 (11)	−0.0055 (12)	−0.0046 (12)
O3	0.0163 (14)	0.0095 (14)	0.0110 (15)	0.0033 (11)	−0.0009 (12)	−0.0023 (12)
O4	0.0136 (14)	0.0115 (15)	0.0166 (16)	0.0057 (11)	0.0004 (12)	−0.0068 (13)
O5	0.0178 (15)	0.0141 (15)	0.0081 (15)	−0.0011 (12)	−0.0022 (12)	−0.0036 (12)
O6	0.0125 (14)	0.0143 (15)	0.0122 (15)	−0.0021 (12)	−0.0020 (12)	−0.0035 (13)
O7	0.0194 (15)	0.0101 (15)	0.0114 (15)	0.0023 (12)	−0.0045 (12)	−0.0018 (12)
O8	0.0198 (15)	0.0112 (15)	0.0168 (16)	0.0026 (12)	−0.0108 (13)	−0.0068 (13)
N1	0.0156 (17)	0.0156 (19)	0.0114 (18)	−0.0043 (14)	−0.0009 (15)	−0.0052 (15)
N2	0.0120 (16)	0.0084 (17)	0.0102 (17)	0.0012 (13)	−0.0048 (14)	−0.0016 (14)
N3	0.0143 (17)	0.0085 (17)	0.0107 (18)	0.0027 (13)	−0.0031 (14)	−0.0015 (14)
N4	0.0078 (15)	0.0116 (17)	0.0108 (18)	0.0015 (13)	−0.0038 (13)	−0.0036 (14)
N5	0.0167 (17)	0.0119 (18)	0.0103 (18)	−0.0026 (14)	−0.0027 (14)	−0.0040 (15)
N6	0.0113 (16)	0.0089 (17)	0.0134 (18)	0.0012 (13)	−0.0034 (14)	−0.0038 (15)
N7	0.0133 (17)	0.0120 (18)	0.0113 (18)	−0.0002 (14)	−0.0011 (14)	−0.0006 (15)
N8	0.0115 (16)	0.0092 (17)	0.0062 (17)	0.0006 (13)	−0.0027 (13)	−0.0035 (14)
C1	0.0119 (19)	0.0065 (19)	0.014 (2)	−0.0032 (15)	0.0020 (16)	−0.0034 (17)
C2	0.012 (2)	0.012 (2)	0.019 (2)	0.0002 (16)	−0.0025 (17)	−0.0047 (18)
C3	0.013 (2)	0.008 (2)	0.029 (3)	0.0021 (16)	−0.0006 (19)	−0.0045 (19)
C4	0.017 (2)	0.013 (2)	0.023 (2)	−0.0019 (17)	0.0011 (19)	−0.0111 (19)
C5	0.014 (2)	0.019 (2)	0.019 (2)	−0.0003 (17)	−0.0031 (18)	−0.0071 (19)
C6	0.014 (2)	0.013 (2)	0.017 (2)	0.0007 (16)	−0.0037 (17)	−0.0043 (18)
C7	0.017 (2)	0.013 (2)	0.009 (2)	−0.0015 (16)	−0.0057 (17)	−0.0012 (17)
C8	0.018 (2)	0.017 (2)	0.015 (2)	0.0029 (18)	−0.0051 (18)	−0.0048 (19)
C9	0.019 (2)	0.020 (2)	0.019 (2)	−0.0003 (18)	−0.0003 (19)	−0.008 (2)
C10	0.027 (2)	0.015 (2)	0.013 (2)	−0.0042 (18)	−0.0040 (19)	−0.0032 (19)
C11	0.026 (2)	0.011 (2)	0.020 (2)	0.0025 (18)	−0.012 (2)	−0.0003 (19)
C12	0.018 (2)	0.016 (2)	0.016 (2)	0.0017 (17)	−0.0047 (18)	−0.0039 (19)
C13	0.0126 (19)	0.0095 (19)	0.0068 (19)	0.0035 (15)	−0.0044 (16)	−0.0009 (16)
C14	0.018 (2)	0.014 (2)	0.015 (2)	0.0030 (17)	−0.0068 (18)	−0.0051 (18)
C15	0.015 (2)	0.012 (2)	0.017 (2)	−0.0001 (17)	0.0002 (18)	0.0003 (18)
C16	0.021 (2)	0.016 (2)	0.013 (2)	0.0087 (18)	−0.0036 (18)	−0.0044 (18)
C17	0.022 (2)	0.017 (2)	0.012 (2)	0.0042 (18)	−0.0071 (18)	−0.0058 (18)
C18	0.014 (2)	0.014 (2)	0.018 (2)	0.0022 (17)	−0.0028 (18)	−0.0054 (19)
C19	0.0106 (19)	0.008 (2)	0.013 (2)	0.0004 (15)	−0.0021 (16)	−0.0019 (17)
C20	0.0118 (19)	0.017 (2)	0.012 (2)	−0.0029 (17)	−0.0001 (17)	−0.0024 (18)
C21	0.015 (2)	0.018 (2)	0.026 (3)	−0.0024 (18)	−0.0021 (19)	−0.009 (2)
C22	0.015 (2)	0.014 (2)	0.026 (3)	0.0024 (17)	−0.0041 (19)	−0.003 (2)
C23	0.018 (2)	0.016 (2)	0.016 (2)	0.0034 (18)	−0.0040 (18)	−0.0015 (19)
C24	0.0130 (19)	0.014 (2)	0.014 (2)	0.0016 (16)	−0.0029 (17)	−0.0064 (18)

### Geometric parameters (Å, º)

**Table:**

Pb1—O1	2.464 (3)	C5—H5	0.9500
Pb1—O1^i^	2.599 (3)	C6—H6	0.9500
Pb1—O2	2.475 (3)	C7—C12	1.372 (6)
Pb1—O3	2.341 (3)	C7—C8	1.389 (6)
Pb1—O4	2.410 (3)	C8—C9	1.397 (6)
Pb2—O5	2.385 (3)	C8—H8	0.9500
Pb2—O6	2.446 (3)	C9—C10	1.396 (6)
Pb2—O7	2.393 (3)	C9—H9	0.9500
Pb2—O7^ii^	2.943 (3)	C10—C11	1.371 (6)
Pb2—O8	2.340 (3)	C10—H10	0.9500
O1—N1	1.297 (4)	C11—C12	1.390 (6)
O1—Pb1^i^	2.599 (3)	C11—H11	0.9500
O2—N2	1.316 (4)	C12—H12	0.9500
O3—N3	1.315 (4)	C13—C18	1.385 (5)
O4—N4	1.303 (4)	C13—C14	1.386 (6)
O5—N5	1.308 (4)	C14—C15	1.388 (6)
O6—N6	1.304 (4)	C14—H14	0.9500
O7—N7	1.306 (4)	C15—C16	1.388 (6)
O8—N8	1.308 (4)	C15—H15	0.9500
N1—N2	1.291 (5)	C16—C17	1.381 (6)
N2—C1	1.452 (5)	C16—H16	0.9500
N3—N4	1.277 (4)	C17—C18	1.388 (6)
N4—C7	1.464 (5)	C17—H17	0.9500
N5—N6	1.290 (5)	C18—H18	0.9500
N6—C13	1.444 (5)	C19—C24	1.381 (6)
N7—N8	1.289 (5)	C19—C20	1.383 (6)
N8—C19	1.446 (5)	C20—C21	1.394 (6)
C1—C6	1.375 (6)	C20—H20	0.9500
C1—C2	1.390 (5)	C21—C22	1.387 (6)
C2—C3	1.393 (6)	C21—H21	0.9500
C2—H2	0.9500	C22—C23	1.379 (6)
C3—C4	1.376 (6)	C22—H22	0.9500
C3—H3	0.9500	C23—C24	1.389 (6)
C4—C5	1.383 (6)	C23—H23	0.9500
C4—H4	0.9500	C24—H24	0.9500
C5—C6	1.395 (6)		
			
O3—Pb1—O4	65.01 (9)	C4—C5—H5	119.7
O3—Pb1—O1	74.85 (9)	C6—C5—H5	119.7
O4—Pb1—O1	139.49 (9)	C1—C6—C5	118.6 (4)
O3—Pb1—O2	78.49 (9)	C1—C6—H6	120.7
O4—Pb1—O2	112.69 (10)	C5—C6—H6	120.7
O1—Pb1—O2	61.82 (9)	C12—C7—C8	123.1 (4)
O3—Pb1—O1^i^	76.97 (10)	C12—C7—N4	118.0 (4)
O4—Pb1—O1^i^	99.80 (9)	C8—C7—N4	119.0 (4)
O1—Pb1—O1^i^	64.69 (11)	C7—C8—C9	117.3 (4)
O2—Pb1—O1^i^	125.15 (9)	C7—C8—H8	121.3
O8—Pb2—O5	76.10 (10)	C9—C8—H8	121.3
O8—Pb2—O7	65.62 (9)	C10—C9—C8	120.6 (4)
O5—Pb2—O7	73.49 (9)	C10—C9—H9	119.7
O8—Pb2—O6	100.77 (10)	C8—C9—H9	119.7
O5—Pb2—O6	63.59 (9)	C11—C10—C9	119.7 (4)
O7—Pb2—O6	137.04 (9)	C11—C10—H10	120.1
O8—Pb2—O7^ii^	119.66 (9)	C9—C10—H10	120.1
O5—Pb2—O7^ii^	118.21 (9)	C10—C11—C12	121.0 (4)
O7—Pb2—O7^ii^	64.60 (10)	C10—C11—H11	119.5
O6—Pb2—O7^ii^	139.15 (8)	C12—C11—H11	119.5
N1—O1—Pb1	120.9 (2)	C7—C12—C11	118.2 (4)
N1—O1—Pb1^i^	115.5 (2)	C7—C12—H12	120.9
Pb1—O1—Pb1^i^	115.31 (11)	C11—C12—H12	120.9
N2—O2—Pb1	114.4 (2)	C18—C13—C14	121.1 (4)
N3—O3—Pb1	121.5 (2)	C18—C13—N6	118.0 (3)
N4—O4—Pb1	114.3 (2)	C14—C13—N6	121.0 (4)
N5—O5—Pb2	123.0 (2)	C13—C14—C15	119.0 (4)
N6—O6—Pb2	115.2 (2)	C13—C14—H14	120.5
N7—O7—Pb2	118.8 (2)	C15—C14—H14	120.5
N8—O8—Pb2	115.6 (2)	C14—C15—C16	120.4 (4)
N2—N1—O1	113.1 (3)	C14—C15—H15	119.8
N1—N2—O2	124.0 (3)	C16—C15—H15	119.8
N1—N2—C1	117.5 (3)	C17—C16—C15	119.9 (4)
O2—N2—C1	118.5 (3)	C17—C16—H16	120.1
N4—N3—O3	114.2 (3)	C15—C16—H16	120.1
N3—N4—O4	124.3 (3)	C16—C17—C18	120.3 (4)
N3—N4—C7	118.0 (3)	C16—C17—H17	119.8
O4—N4—C7	117.6 (3)	C18—C17—H17	119.8
N6—N5—O5	113.3 (3)	C13—C18—C17	119.3 (4)
N5—N6—O6	124.3 (3)	C13—C18—H18	120.4
N5—N6—C13	117.3 (3)	C17—C18—H18	120.4
O6—N6—C13	118.4 (3)	C24—C19—C20	122.3 (4)
N8—N7—O7	114.1 (3)	C24—C19—N8	117.3 (4)
N7—N8—O8	124.5 (3)	C20—C19—N8	120.4 (4)
N7—N8—C19	117.7 (3)	C19—C20—C21	117.8 (4)
O8—N8—C19	117.8 (3)	C19—C20—H20	121.1
C6—C1—C2	122.2 (4)	C21—C20—H20	121.1
C6—C1—N2	118.5 (4)	C22—C21—C20	121.1 (4)
C2—C1—N2	119.3 (4)	C22—C21—H21	119.4
C1—C2—C3	117.8 (4)	C20—C21—H21	119.4
C1—C2—H2	121.1	C23—C22—C21	119.4 (4)
C3—C2—H2	121.1	C23—C22—H22	120.3
C4—C3—C2	121.2 (4)	C21—C22—H22	120.3
C4—C3—H3	119.4	C22—C23—C24	120.8 (4)
C2—C3—H3	119.4	C22—C23—H23	119.6
C3—C4—C5	119.7 (4)	C24—C23—H23	119.6
C3—C4—H4	120.2	C19—C24—C23	118.6 (4)
C5—C4—H4	120.2	C19—C24—H24	120.7
C4—C5—C6	120.6 (4)	C23—C24—H24	120.7
			
O3—Pb1—O1—N1	−64.4 (3)	O7—N7—N8—O8	−0.3 (5)
O4—Pb1—O1—N1	−72.2 (3)	O7—N7—N8—C19	−179.6 (3)
O2—Pb1—O1—N1	20.4 (2)	Pb2—O8—N8—N7	−9.0 (4)
O1^i^—Pb1—O1—N1	−146.9 (3)	Pb2—O8—N8—C19	170.4 (2)
O3—Pb1—O1—Pb1^i^	82.51 (12)	N1—N2—C1—C6	168.1 (4)
O4—Pb1—O1—Pb1^i^	74.70 (18)	O2—N2—C1—C6	−12.6 (5)
O2—Pb1—O1—Pb1^i^	167.38 (15)	N1—N2—C1—C2	−13.4 (5)
O1^i^—Pb1—O1—Pb1^i^	0.0	O2—N2—C1—C2	165.9 (3)
O3—Pb1—O2—N2	60.2 (2)	C6—C1—C2—C3	−1.5 (6)
O4—Pb1—O2—N2	116.7 (2)	N2—C1—C2—C3	180.0 (4)
O1—Pb1—O2—N2	−18.6 (2)	C1—C2—C3—C4	0.1 (6)
O1^i^—Pb1—O2—N2	−4.6 (3)	C2—C3—C4—C5	0.4 (6)
O4—Pb1—O3—N3	−7.0 (2)	C3—C4—C5—C6	0.6 (6)
O1—Pb1—O3—N3	178.6 (3)	C2—C1—C6—C5	2.4 (6)
O2—Pb1—O3—N3	115.0 (3)	N2—C1—C6—C5	−179.0 (4)
O1^i^—Pb1—O3—N3	−114.5 (3)	C4—C5—C6—C1	−2.0 (6)
O3—Pb1—O4—N4	6.2 (2)	N3—N4—C7—C12	167.6 (4)
O1—Pb1—O4—N4	14.5 (3)	O4—N4—C7—C12	−9.8 (5)
O2—Pb1—O4—N4	−58.1 (3)	N3—N4—C7—C8	−11.3 (6)
O1^i^—Pb1—O4—N4	76.7 (2)	O4—N4—C7—C8	171.4 (4)
O8—Pb2—O5—N5	−115.5 (3)	C12—C7—C8—C9	−2.2 (7)
O7—Pb2—O5—N5	176.3 (3)	N4—C7—C8—C9	176.6 (4)
O6—Pb2—O5—N5	−5.7 (3)	C7—C8—C9—C10	0.5 (7)
O7^ii^—Pb2—O5—N5	128.1 (3)	C8—C9—C10—C11	1.1 (7)
O8—Pb2—O6—N6	74.2 (2)	C9—C10—C11—C12	−1.2 (7)
O5—Pb2—O6—N6	5.7 (2)	C8—C7—C12—C11	2.1 (7)
O7—Pb2—O6—N6	8.5 (3)	N4—C7—C12—C11	−176.7 (4)
O7^ii^—Pb2—O6—N6	−97.7 (3)	C10—C11—C12—C7	−0.3 (7)
O8—Pb2—O7—N7	−10.1 (2)	N5—N6—C13—C18	−169.3 (4)
O5—Pb2—O7—N7	71.8 (2)	O6—N6—C13—C18	8.8 (5)
O6—Pb2—O7—N7	69.2 (3)	N5—N6—C13—C14	11.6 (5)
O7^ii^—Pb2—O7—N7	−154.8 (3)	O6—N6—C13—C14	−170.3 (3)
O5—Pb2—O8—N8	−68.8 (2)	C18—C13—C14—C15	0.4 (6)
O7—Pb2—O8—N8	9.1 (2)	N6—C13—C14—C15	179.4 (4)
O6—Pb2—O8—N8	−127.9 (2)	C13—C14—C15—C16	−0.5 (6)
O7^ii^—Pb2—O8—N8	46.0 (3)	C14—C15—C16—C17	0.9 (6)
Pb1—O1—N1—N2	−18.8 (4)	C15—C16—C17—C18	−1.0 (6)
Pb1^i^—O1—N1—N2	−165.7 (2)	C14—C13—C18—C17	−0.5 (6)
O1—N1—N2—O2	−1.3 (5)	N6—C13—C18—C17	−179.5 (3)
O1—N1—N2—C1	178.0 (3)	C16—C17—C18—C13	0.8 (6)
Pb1—O2—N2—N1	19.4 (4)	N7—N8—C19—C24	160.1 (3)
Pb1—O2—N2—C1	−159.8 (2)	O8—N8—C19—C24	−19.3 (5)
Pb1—O3—N3—N4	6.8 (4)	N7—N8—C19—C20	−18.8 (5)
O3—N3—N4—O4	−0.2 (5)	O8—N8—C19—C20	161.8 (3)
O3—N3—N4—C7	−177.4 (3)	C24—C19—C20—C21	−1.7 (6)
Pb1—O4—N4—N3	−5.9 (4)	N8—C19—C20—C21	177.1 (3)
Pb1—O4—N4—C7	171.3 (2)	C19—C20—C21—C22	0.9 (6)
Pb2—O5—N5—N6	4.8 (4)	C20—C21—C22—C23	0.4 (6)
O5—N5—N6—O6	1.5 (5)	C21—C22—C23—C24	−0.8 (6)
O5—N5—N6—C13	179.4 (3)	C20—C19—C24—C23	1.3 (6)
Pb2—O6—N6—N5	−6.4 (5)	N8—C19—C24—C23	−177.6 (3)
Pb2—O6—N6—C13	175.6 (2)	C22—C23—C24—C19	0.0 (6)
Pb2—O7—N7—N8	9.5 (4)		

Symmetry codes: (i) −*x*+2, −*y*+1, −*z*+1; (ii) −*x*+1, −*y*+1, −*z*+1.
